# Supplementary material for: Hierarchical zwitterionic modification of a SERS substrate enables real-time drug monitoring in blood plasma
Source: Nat Commun. 2016 Nov 11;7:13437. doi: 10.1038/ncomms13437 (PMC5114600; doi:10.1038/ncomms13437)
Supplement: Supplementary Information — Supplementary Figures 1-7, Supplementary Tables 1-4, Supplementary Methods and Supplementary Reference. [file ncomms13437-s1.pdf]

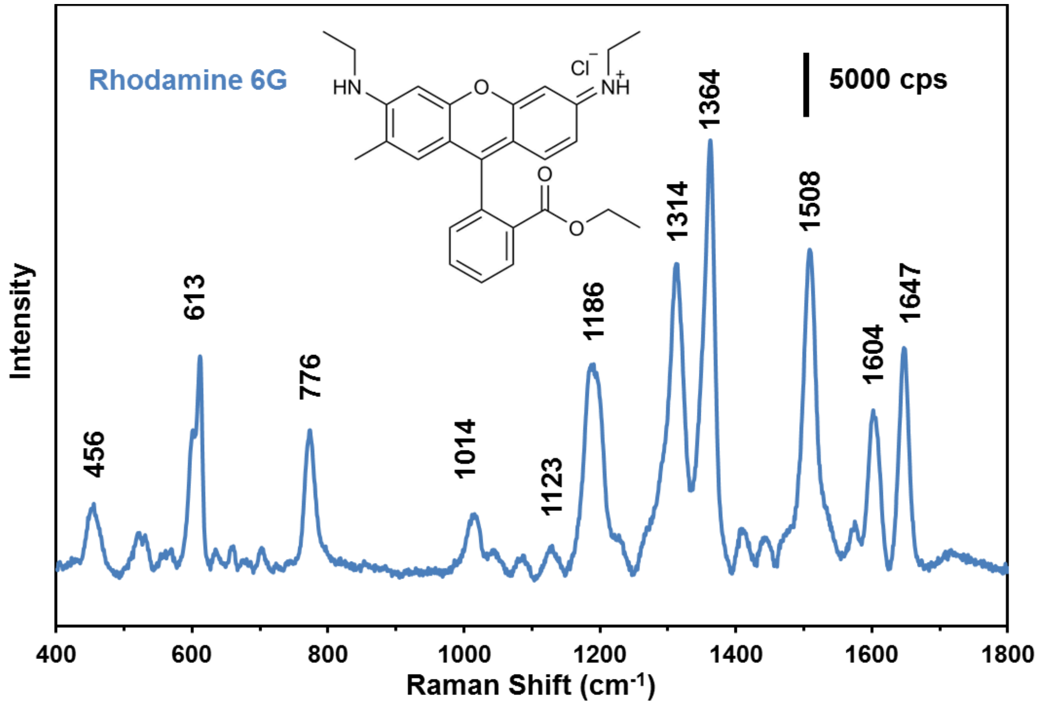

Supplementary Figure 1. SERS spectrum of 1 µM R6G in DI water on a bare gold Q3D-PNA SERS substrate. The λ_{ex} = 785 nm, P_{laser} = 1 mW, and t = 30 s with 3 accumulations.

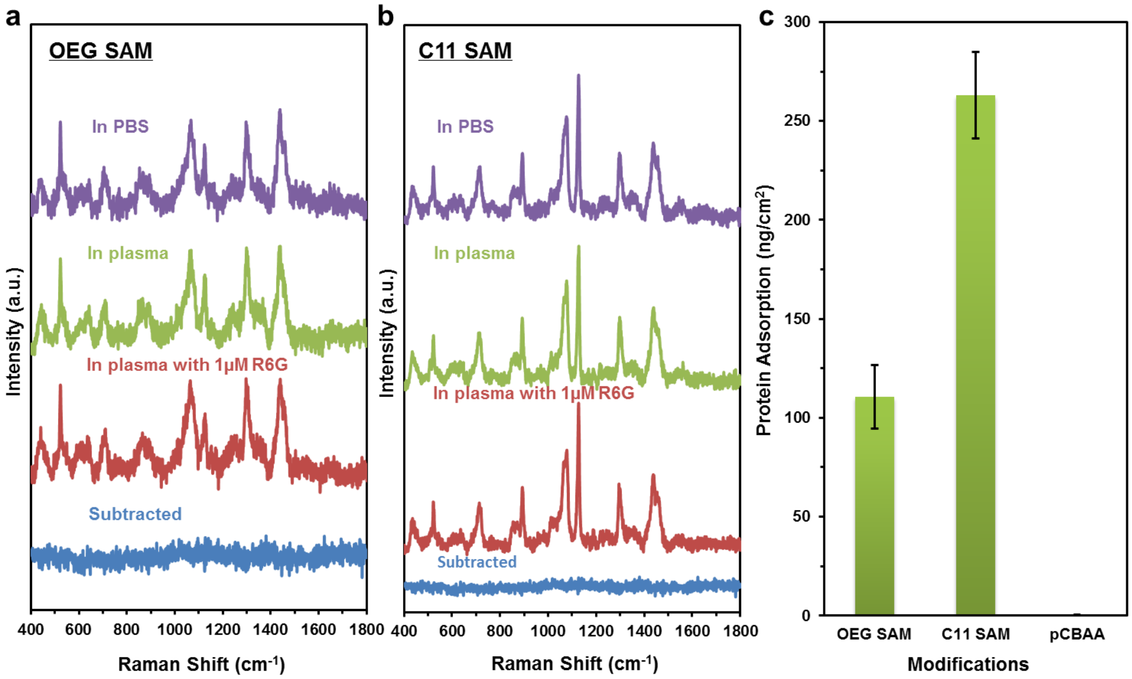

Supplementary Figure 2. OEG SAM and C11 SAM modified SERS sensors challenged with undiluted human blood plasma. a,b, SERS spectra acquired with the OEG SAM-modified and C11 SAM-modified SERS optofluidic systems. From top to bottom, spectra shown were recorded after flowing PBS, undiluted plasma, and plasma containing 1 μM R6G each for 10 min, along with the subtracted spectrum from the last two. λ_{ex} = 785 nm, P_{laser} = 1 mW, and t = 30 s with 3 accumulations. **c**, Protein adsorption from undiluted plasma on OEG SAM and C11 SAM-modified gold surfaces, measured by an SPR biosensor. The error bar represent the standard deviation of three replicates.

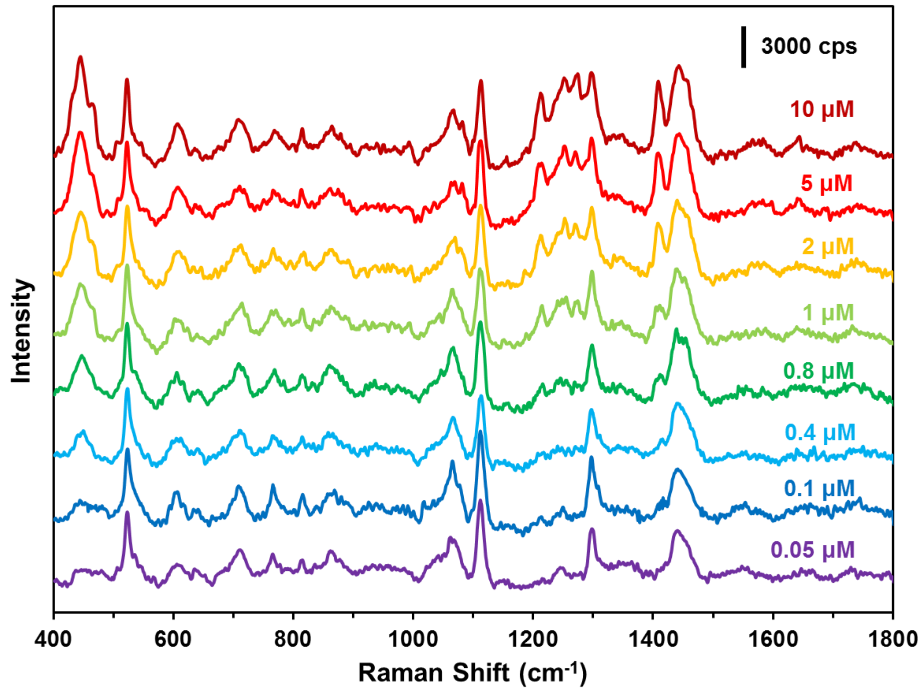

Supplementary Figure 3. SERS spectra of DOX in plasma UF control at concentrations ranging from 0.05 to 10 μM. λ_{ex} = 785 nm, P_{laser} = 1 mW, and t = 30 s with 3 accumulations.

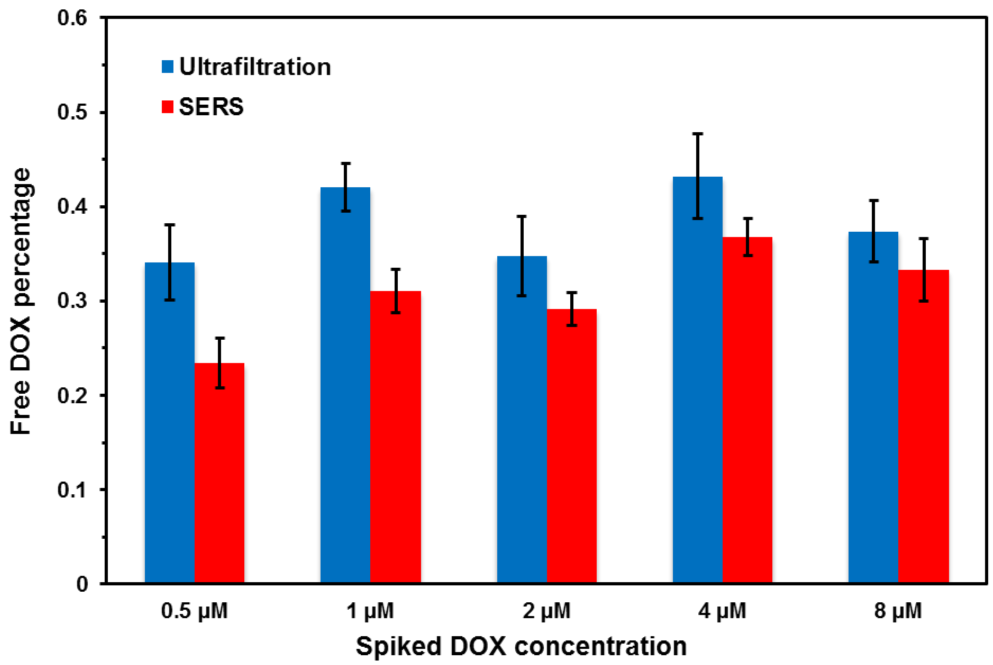

Supplementary Figure 4. Free DOX percentages in plasma measured by ultrafiltration and SERS methods. Error bars represent the standard deviation of three replicates.

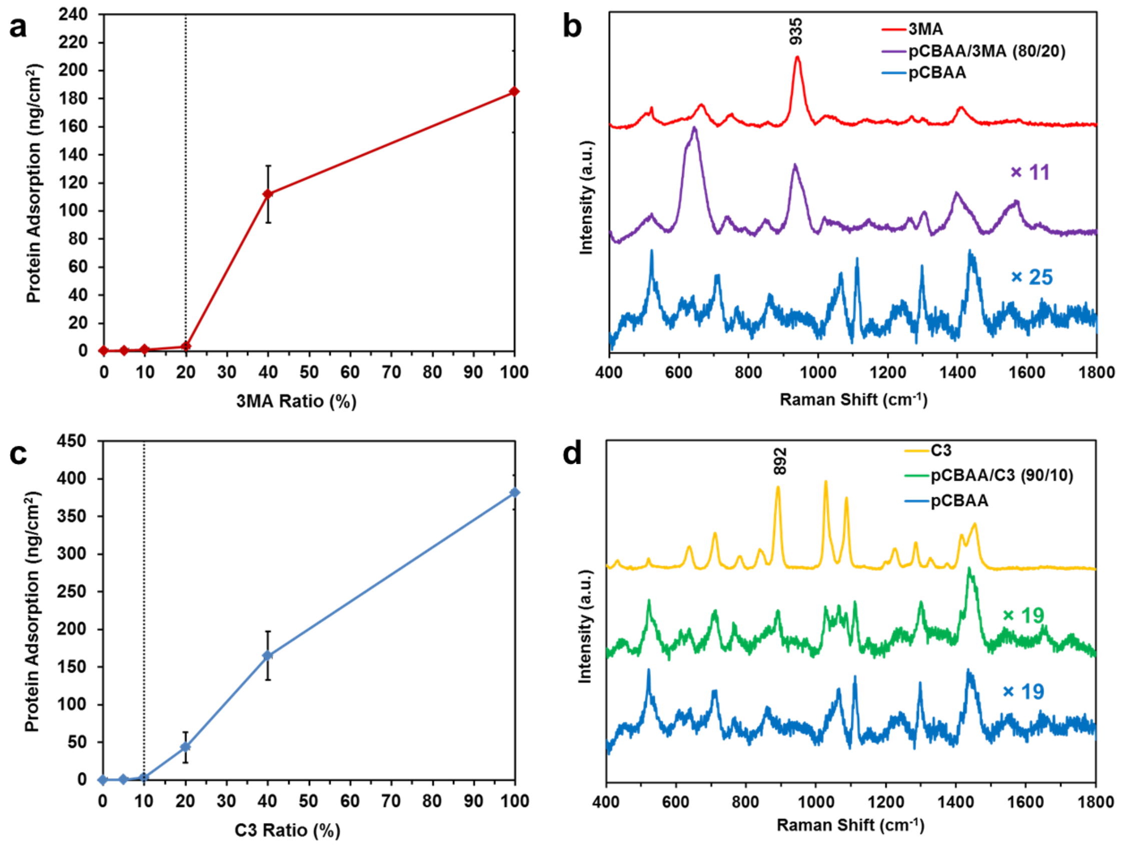

Supplementary Figure 5. a, Protein adsorption from human blood plasma on hierarchical pCBAA modified gold surfaces as a function of 3MA ratio (in stock solution) measured by SPR, showing that the excellent non-fouling property (< 5 ng/cm^2) can be maintained down to an 80/20 initiator/3MA ratio in stock solution. **b,** SERS spectra of a pure 3MA SAM, a hierarchical pCBAA film with the SAM layer of initiator/3MA (80/20, in stock solution) and a pCBAA film grafted from a pure initiator SAM. Based on the absolute intensities of the peak at 935 cm^{-1} from the pure 3MA SAM (red line) and initiator/3MA mixed SAM (purple line), the ratio of initiator/3MA mixed SAM is 92/8 on the SERS surface. **c,** Protein adsorption from human blood plasma on hierarchical pCBAA modified gold surfaces as a function of C3 ratio (in stock solution) measured by SPR, showing that the excellent non-fouling property (< 5 ng/cm^2) can be maintained down to a 90/10 initiator/C3 ratio in stock solution. **d,** SERS spectra of a pure C3 SAM, a hierarchical pCBAA film with an initiator/C3 SAM layer (90/10, in stock solution) and a pCBAA film grafted from a pure initiator SAM. Based on the absolute peak intensities at 892 cm^{-1} from the pure C3 SAM (yellow line) and initiator/C3 mixed SAM (green line), the ratio of initiator:C3 in the mixed SAM is 95/5 on the SERS surface. The error bars in a and c represent standard deviations of three replicates.

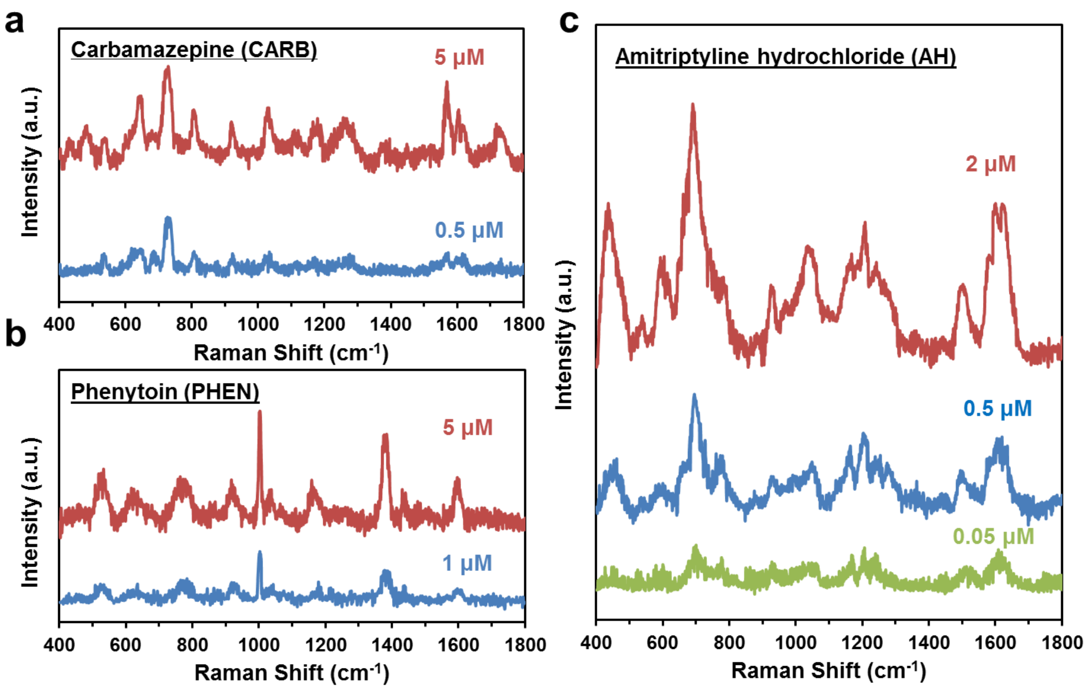

Supplementary Figure 6. Limit of detection (LOD) experiments for CARB (a), PHEN (b) and AH (c), using the hierarchical pCBAA-modified SERS optofluidic system.

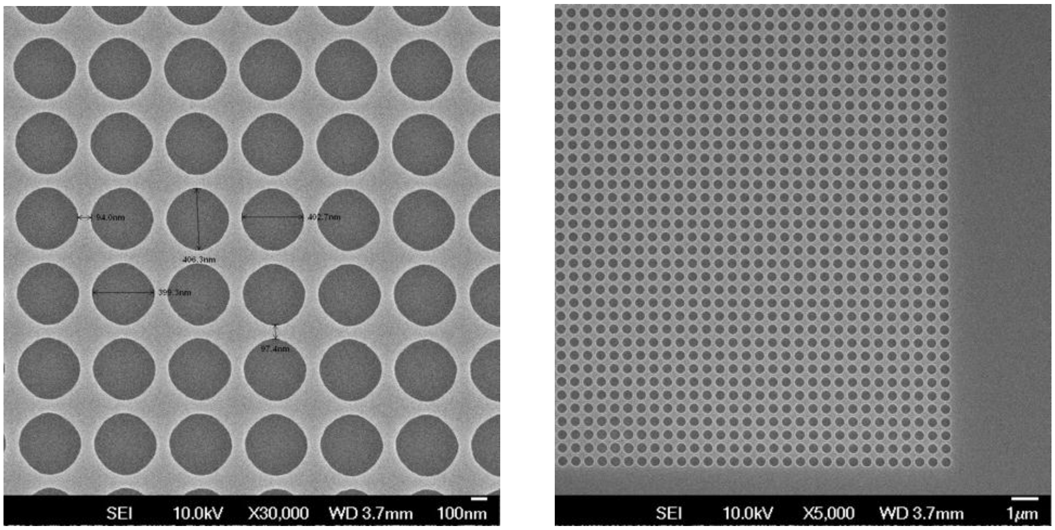

Supplementary Figure 7. SEM images of Q3D-PNAs SERS substrate, showing the precisely fabricated gold nanoholes array. The uniformity of nanoholes from corner to center was ensured by the high-end EBL instrument used.

Supplementary Table 1. SERS vibrational frequencies for 100% human blood plasma

Peak position (cm^{-1})	Vibrational mode [a]	Assignments
482	ν(S-S)	L-Arginine
578		Ascorbic acid or cholesterol
631	β (C-C)	Uric acid
724	β(C-H)	Hypoxanthine or adenine
754	β(C-H)	Adenine
838	ν(C-C-O)	L-Serine
881	ν(C-C)	Uric acid
955	ν(C-C)	Hypoxanthine or adenine
1036	ν(C-N)	Uric acid
1138	ν(C-N)	Uric acid
1325	ν(C-H)	Nucleic acid bases
1360	β(CH_3)	Adenine
1445	β(CH_2)	Collagen, phospholipids
1580	β(C=C)	Phenylalanine
1649	ν(C=O)	Amide I

[a] ν; stretching, β; bending

Supplementary Table 2. SERS vibrational frequencies for pCBAA modification

**Peak position (cm^{-1})**	**Vibrational mode** [a]
520	Si
638	$\nu(C\text{-}S)_G$
710	$\nu(C\text{-}S)_T$
764	$\beta(C\text{-}H)$
864	CH_2 rock
881	CH_3 rock
1066	$\nu(C\text{-}C)_T$
1112	$\nu(C\text{-}C)_T$
1244	$\nu(C\text{-}C)_t$
1441	$\beta(CH_2)$
1649	Amide I

[a] ν; stretching, β; bending

Supplementary Table 3. Film thickness of pure and mixed SAMs measured by ellipsometry and the molar ratio of mixed SAMs estimated from the film thicknesses.

	**Initiator (I)**	**3MA**	**C3**	**I/3MA (90/10)[a]**	**I/3MA (80/20)[a]**	**I/C3 (90/10)[a]**	**I/C3 (80/20)[a]**
**Thickness (Å)**	22.4 ± 0.7	10.4 ± 0.5	9.3 ± 0.3	21.4 ± 0.3	21.0 ± 0.4	21.5 ± 0.2	20.4 ± 0.5
**Surface Ratio[b]**				92/8	89/11	93/7	85/15

[a]The molar ratio of initiator/short thiol in bulk solutions prepared to form mixed SAMs.
[b]The molar ratio of initiator/short thiol in mixed SAMs, calculated from the ellipsometric thicknesses.

Supplementary Table 4. Elemental composition and surface ratio of mixed SAMs analyzed by XPS.

	**Elemental Composition (%)**					**Surface Ratio**	
	**Br 3p**	**S 2p**	**C 1s**	**O 1s**	**Au 4f**	**Method [b]**	**Method [c]**
**I/3MA (80/20)[a]**	2.4 ± 0.3	2.7 ± 0.2	38.4 ± 1.4	5.6 ± 0.3	50.9 ± 2.1	90/10	93/7
**I/C3 (90/10)[a]**	2.5 ± 0.2	2.7 ± 0.3	39.0 ± 2.2	5.1 ± 0.5	50.6 ± 1.7	93/7	95/5

[a]The molar ratio of initiator/short thiol in bulk solutions prepared for forming mixed SAMs.
[b]The ratio of initiator/short thiol in mixed SAMs, calculated based on the Br/S elemental ratio.
[c]The ratio of initiator/short thiol in mixed SAMs, calculated based on the C/S elemental ratio.

Supplementary Methods

Study of OEG and C11 SAM modified SERS optofluidic systems

The OEG thiol ($HS(CH_2)_{11}(OCH_2)_4OH$) and 1-Undecanethiol ($HS(CH_2)_{10}CH_3$ C11 thiol) SAMs were formed on UV-ozone cleaned SERS substrates and SPR chips by soaking bare substrates or chips overnight in a 1 mM solution containing the corresponding thiol in pure ethanol. Using the same SERS optofluidic system used in other tests, four solutions were pumped over the OEG SAM or C11 SAM-modified SERS substrates in succession: 1) saline buffer (PBS); 2) plasma; 3) PBS again; and 4) plasma spiked with 1 μM R6G. Each solution was circulated for 10 min, and SERS spectra were continuously collected. The nonspecific protein adsorption from undiluted plasma onto OEG and C11 SAMs were measured by an SPR sensor.

Determination of free DOX in plasma using ultrafiltration

A range of DOX concentrations (0.5, 1, 2, 4 and 8 μM) were investigated to determine the plasma binding parameters. Experiments for each concentration were run in triplicate and results were expressed as the mean ± standard deviation. To establish a standard curve, plasma ultrafiltrate (UF) control matrix was prepared from human plasma using an Amicon ultra-centrifugal filter device (10 kDa) centrifuged at 2000× g RCF for 30 min. UF calibration standards were prepared by adding 30 μL of working standards into 270 μL of control UF to provide final DOX concentrations in UF ranging from 0.2 to 4 μM; then, 90 μL of 1 ng/mL DOX was added as an internal standard (IS) to a 30 μL aliquot of each UF standard for analysis. Sciex 400Qtrap LC-MS/MS system with Turbo IonSpray (Applied Biosystems) operating in positive ion mode was adopted for compound detection. DOX working solution was mixed with human plasma for final concentrations from 0.5 to 8 μM (the same samples as those used for SERS detection). After 1 h incubation, 1 mL portions of human plasma solutions supplemented with various concentrations of DOX were placed in ultra-centrifugal filter, and ultrafiltrate containing free DOX was obtained by subjecting the system to centrifugation at 2000 × g RCF for 30 min. Analytical recovery of DOX in this system was about 95%. A 100 μL aliquot of the ultrafiltrate containing free DOX was subjected to LC-MS analysis and the concentration of free drug was determined based on the standard curve. The percentage of free DOX in plasma was determined as follows: F/T × 100, where F denotes the free-drug concentration determined in the ultrafiltration and T is the total drug concentration.

Estimation of surface composition of mixed SAMs based on SERS spectral intensities

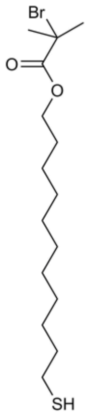

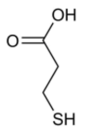

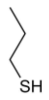

Initiator (I)   3-mercaptopropionic acid (3MA)   1-propanethiol (C3)

It is known that the final surface composition ratio of two components forming a SAM differs from their ratio in the SAM-forming bulk solution. We first estimated the surface ratio of initiator:3MA by taking the ratio of the absolute intensities of the 935 cm^{-1} peak, which is attributed solely to 3MA. We assumed equivalent total surface coverage of pure 3MA and initiator/3MA mixed SAMs, and that one initiator molecule provides surface coverage equivalent to one 3MA molecule on the SERS substrate. In this way, a change in the peak intensity at 935 cm^{-1} is directly attributed to a change in the number of 3MA molecules on the surface. On the basis of this assumption, we deduced that the initiator:3MA surface ratio in mixed SAMs was 92:8, corresponding to a molar ratio of 80:20 in the bulk solution. The higher fraction of initiator molecules in the SAMs may be due to stronger interactions between their longer alkane chains, which make initiator SAMs more stable than those formed from the short alkanethiol. Similarly, the surface ratio of initiator:C3 in mixed SAMs was 95:5, corresponding to a molar ratio of 90:10 in bulk solutions, based on the peak intensities at 892 cm^{-1}.

Estimation of surface composition of mixed SAMs based on ellipsometry film thicknesses

Surface compositions of mixed SAMs were also estimated from the film thicknesses measured by ellipsometry. The thicknesses of the single-component initiator SAM, 3MA SAM, and C3 SAM as well as the mixed initiator/3MA and initiator/C3 SAMs were determined using ellipsometry, (Model alpha-SE, J.A. Woollam, Lincoln, NE) in a 380–900 nm wavelength range at an incidence angle of 70°. The results were fitted to Cauchy model. Three locations on each sample were analyzed. To determine the molar ratio of initiator/short thiol (3MA or C3) on the surface, we assumed that the measured ellipsometric thickness of a mixed SAM, d_{mixed}, is directly related to the surface composition through the equation: $d_{\text{mixed}} = \chi_{\text{intiator}}\, d_{\text{initator}} + \chi_{\text{short thiol}}\, d_{\text{short thiol}}$, where χ_{intiator} and $\chi_{\text{short thiol}}$ are the mole fractions of initiator and short thiol in the mixed SAM, respectively, and $\chi_{\text{intiator}} + \chi_{\text{short thiol}} = 1$; d_{initator} and $d_{\text{short thiol}}$ are the thicknesses of single-component initiator SAM and short thiol SAM measured by ellipsometry, respectively. This assumption requires the refraction indices of pure and mixed SAMs to be the same. [1]

Estimation of surface composition of mixed SAMs based on XPS analysis

The initiator/3MA and initiator/C3 mixed SAMs were additionally characterized using X-ray photoelectron spectroscopy (XPS) on an Axis Ultra XPS instrument (Kratos) using monochromated Al Kα radiation (1486.6 eV). Survey spectra and detail scans of Br 3p, S 2p, C 1s, O 1s and Au 4f were acquired using a pass energy of 150 eV. Spectra were collected with the analyzer at 55° with respect to the surface normal to the sample. Typical pressure in the chamber during spectral acquisition was 10^{-9} Torr. Three spots on two or more replicate samples were analyzed. Computer aided surface analysis (CasaXPS) software was used to calculate compositions from the peak areas. To determine the molar ratio of initiator/short thiol in the mixed SAM, we used the Br/S elemental ratio. In a single-component initiator SAM, the ideal Br/S elemental ratio is 1. In the mixed SAM, the Br/S ratio is diluted after mixing with the short thiol, which only contains elemental S but no Br. The C/S ratio was also used to calculate the surface composition of initiator/short thiol in the mixed SAM.

Supplementary Reference

[1] Folkers, J. P., Laibinis, P. E., & Whitesides, G. M. Self-assembled monolayers of alkanethiols on gold: comparisons of monolayers containing mixtures of short-and long-chain constituents with methyl and hydroxymethyl terminal groups. *Langmuir* **8**, 1330-1341 (1993).
